# Inflammatory and Infectious Cutaneous Entities Resembling Cutaneous T-Cell Lymphoma (CTCL): An Integrated Clinicopathological Review

**DOI:** 10.3390/dermatopathology13020023

**Published:** 2026-05-27

**Authors:** Jade Nasser Eldin, Elias El Tayar, Ossama Abbas, Jag Bhawan

**Affiliations:** 1 Dermatology Department, American University of Beirut-Medical Center, Beirut P.O. Box 11-0236, Lebanon; 2 Dermatopathology Section, Department of Dermatology, Boston University School of Medicine, Boston, MA 02118, USA

**Keywords:** Cutaneous T-Cell Lymphoma, mycosis fungoides, pseudolymphomatous

## Abstract

Cutaneous pseudolymphomas are benign reactive lymphoid proliferations that often mimic cutaneous lymphomas both clinically and histologically. A diverse array of inflammatory, infectious, and drug-induced dermatoses can closely resemble cutaneous T-cell lymphomas (CTCLs), particularly mycosis fungoides (MFs), posing significant diagnostic challenges. These mimickers may show histopathological features such as epidermotropism, dense lymphocytic infiltrates, or even clonality, making accurate differentiation crucial to avoid overtreatment. This review endeavors to comprehensively discuss the clinicopathologic features of the various inflammatory and infectious dermatoses that may simulate CTCL, drawing on illustrative examples across disease categories. By highlighting important comparative features and emphasizing the importance of clinicopathologic correlation, this review outlines practical strategies for distinguishing true lymphoma from its inflammatory mimics.

## Simple Summary

Cutaneous pseudolymphomas are non-cancerous skin conditions that can closely resemble skin lymphomas, especially a type called mycosis fungoides, both in appearance and under the microscope. They can be triggered by many causes such as autoimmune diseases, infections, medications, insect bites, or even tattoos. Because they can look very similar to true lymphoma, they can sometimes be misdiagnosed, leading to unnecessary anxiety or treatment. This review explains the main conditions that mimic lymphoma and highlights key clinical and microscopic clues that help doctors tell them apart. These include features like the type of immune cells present, patterns in the skin, and how the condition behaves over time. Understanding these differences is essential to ensure patients receive the correct diagnosis and avoid overtreatment.

## 1. Introduction

Cutaneous pseudolymphomas (CPLs) are benign lymphoid proliferations of the skin that histologically and, at times, clinically resemble cutaneous lymphomas, particularly cutaneous T-cell lymphoma (CTCL). These lesions arise as reactive processes to a wide array of stimuli, including infections, drugs, trauma, autoimmune diseases, and foreign materials such as tattoos, or they may even remain idiopathic [1]. While benign by definition and lacking malignant potential and systemic dissemination, CPLs cannot be differentiated from true lymphoma based on clinical presentation and often exhibit histopathological features that raise suspicion for malignancy.

The diagnostic overlap between pseudolymphomatous inflammatory conditions and CTCL, especially mycosis fungoides (MFs), is well-recognized [2,3]. MF itself is a highly polymorphic disease, with several clinicopathologic variants including lichenoid, spongiotic, psoriasiform, folliculotropic, and zosteriform MF, leading to the frequent misclassification of mimickers that can share features such as band-like lymphocytic infiltrates, epidermotropism, adnexotropism, and even molecular evidence of T-cell clonality [4,5]. In addition, pan–T-cell antigen (CD7) loss or partial loss and predominant CD4+ lymphocytes are not exclusive to lymphoma but may also be observed in inflammatory dermatoses and infection [4,6].

Several autoimmune/inflammatory diseases may closely simulate MF such as lupus erythematosus (LE) [7,8]. Recent studies have highlighted the diagnostic utility of CD123 immunostaining in LE [6,9]. Lichen sclerosus, morphea, and lichen planus are other autoimmune interface dermatoses known to histopathologically mimic CTCL [5,10,11,12]. Syringotropic variants of these conditions further blur the lines with adnexotropic MF [13,14]. Psoriasiform diseases, such as palmoplantar pustular psoriasis (PPPP), can also imitate CTCL/MF [15,16]. Similarly, chronic eczematous dermatoses, such as contact and atopic dermatitis, can display prominent lymphoid infiltrates [17,18,19,20].

Drug-induced pseudolymphomas, particularly fixed drug eruptions (FDEs), may mimic CD8+ epidermotropic CTCL in both immunophenotype and clinical course, making diagnosis particularly challenging [21,22]. Tattoo-related pseudolymphomas, characterized by dense dermal lymphoid infiltrates, often CD4+-predominant, pose another diagnostic trap and have been reported for both black and colored inks [23,24].

Infectious etiologies also comprise a significant proportion of pseudolymphomatous conditions. Cutaneous manifestations of syphilis, molluscum contagiosum, herpes simplex virus, and *Borrelia burgdorferi* infection have all been described as mimicking cutaneous lymphomas [25,26,27,28,29]. These conditions may show dense lymphoid infiltrates with atypical cells and, in some cases, even clonal rearrangement [30]. Fungal infections, though less frequently discussed, also warrant consideration in the differential diagnosis of CTCL mimickers [2].

Because histologic mimickers of MF are not only challenging but also hold serious implications for patient management, a misdiagnosis of lymphoma may lead to overtreatment with systemic therapies, unnecessary psychosocial burden, and increased surveillance. On the other hand, failure to diagnose CTCL/MF will delay diagnosis and proper therapy until later [4,6]. Therefore, a high index of suspicion, clinicopathologic correlation, and, when necessary, molecular and immunohistochemical adjuncts are indispensable to ensure diagnostic accuracy. This review aims to provide a comprehensive overview of cutaneous inflammatory and infectious conditions that histologically mimic CTCL/MF. Each entity is described with attention to clinical–pathologic hallmarks and distinguishing features.

## 2. Cutaneous Lupus Erythematous (CLE)

**Figure 1:**
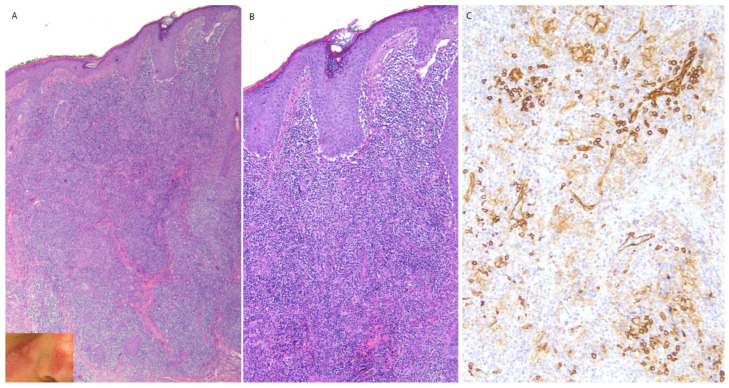
(**A**–**C**) Pseudolymphomatous lupus erythrematosus with CD123-positive pDC clustering. Inset showing the clinical presentation with cheek, nose, and ear involvement. This figure is from the corresponding author’s laboratory and illustrates the pathological concept of typical pseudolymphomatous LE.

Cutaneous lupus erythematosus may rarely present with pseudolymphomatous lymphoid infiltrates that closely mimic CTCL across multiple clinical subtypes including discoid lupus, lupus tumidus, lupus panniculitis, and mucosal lupus (Figure 1) [8,9,31].

Recognition of CD123-positive pDC clustering, in conjunction with dermal mucin, interface change, and preserved T-cell immunophenotype, is critical for distinguishing pseudolymphomatous CLE from true CTCL and avoiding misdiagnosis (Table 1) [6,9,31,32,33].

## 3. Lichen Sclerosus (LS)

**Figure 2:**
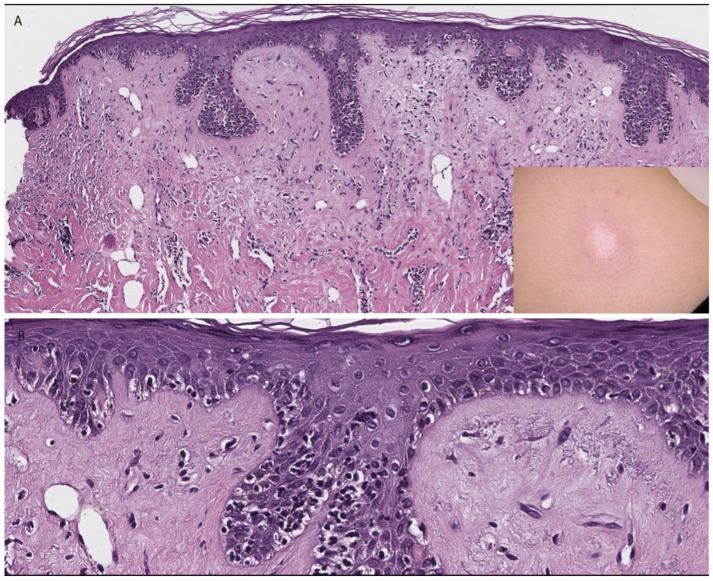
(**A**,**B**) Pseudolymphomatous lichen sclerosus. Inset showing the clinical presentation. This figure is from the corresponding author’s laboratory and illustrates the pathological concept of typical pseudolymphomatous lichen sclerosus.

Lichen sclerosus is a chronic inflammatory dermatosis that may, particularly in early or atypical stages, exhibit pseudolymphomatous histopathologic features closely simulating CTCL, most notably MF, leading to frequent diagnostic confusion (Figure 2) [3,5,10,34].

Early lichen sclerosus is characterized by a lichenoid lymphocytic infiltrate associated with papillary dermal hyalinization and basal vacuolar alteration in the absence of significant cytologic atypia, cerebriform lymphocytes, or disproportionate epidermotropism; involvement of the anogenital region further supports a reactive lichenoid dermatosis rather than mycosis fungoides (Table 2) [3,5,10,34,35].

Notably, several studies have demonstrated that lichen sclerosus may exhibit monoclonal T-cell receptor (TCR) gene rearrangements, including γ-chain clonality, despite its benign nature, underscoring that clonality alone does not establish a diagnosis of lymphoma and must be interpreted in the appropriate clinicopathologic context [36,37,38].

## 4. Vitiligo

**Figure 3:**
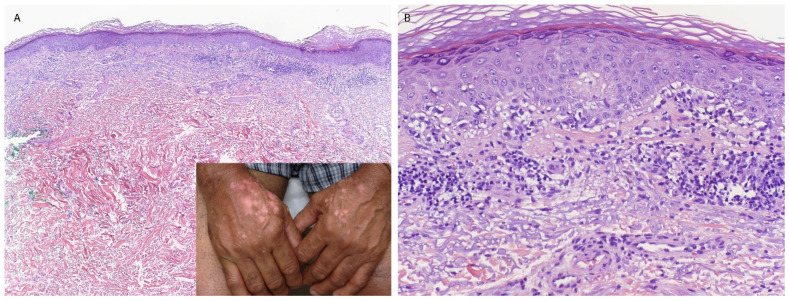
(**A**,**B**) Pseudolymphomatous vitiligo (inset highlights the clinical presentation with depigmented patches on hands). This figure is from the corresponding author’s laboratory and illustrates the pathological concept of typical pseudolymphomatous vitiligo.

Vitiligo is an immune-mediated depigmenting disorder that, in its inflammatory or early stages, may demonstrate pseudolymphomatous histopathologic features mimicking hypopigmented MF, creating a well-recognized diagnostic pitfall (Figure 3) [3,39,40].

The correlation between melanocyte loss, preserved T-cell immunophenotype, type I interferon-associated pDC activity (which promotes local immune activation and recruitment of cytotoxic T cells targeting melanocytes), and clinical evolution is essential for distinguishing pseudolymphomatous vitiligo from hypopigmented MF (Table 3) [3,39,40].

## 5. Pigmented Purpuric Dermatoses/Lichen Aureus

**Figure 4:**
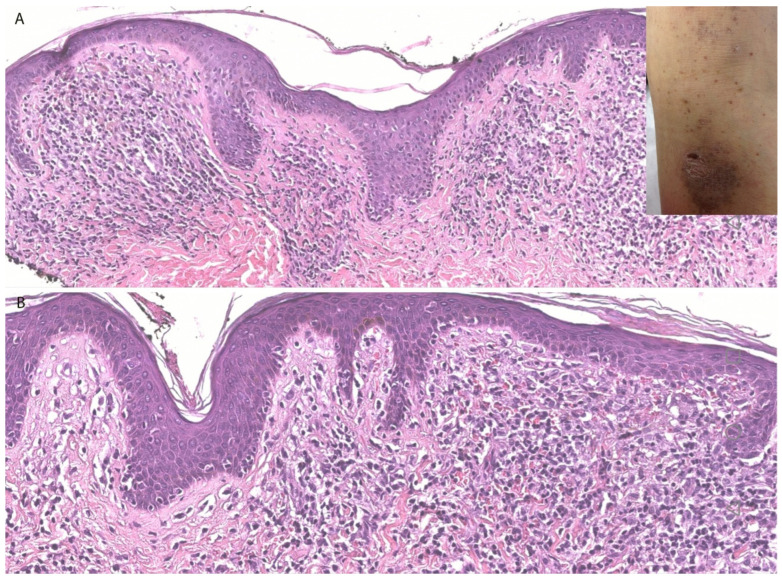
(**A**,**B**) Pigmented purpuric dermatosis. Histology exhibits dense focally band-like dermal lymphoid infiltrate with extravasated erythrocytes. Inset showing the clinical presentation. This figure is from the corresponding author’s laboratory and illustrates the pathological concept of typical pseudolymphomatous PPD.

Pigmented purpuric dermatoses (PPD), particularly lichen aureus, may exhibit pseudolymphomatous lymphoid infiltrates that closely mimic CTCL, especially unilesional or purpuric variants of MF, creating a recognized diagnostic pitfall (Figure 4) [41,42,43].

Recognition of erythrocyte extravasation, hemosiderin deposition, clinical stability, and absence of significant cytologic atypia is essential for distinguishing pseudolymphomatous PPD/lichen aureus from CTCL, even in cases demonstrating partial antigen loss or monoclonal T-cell receptor rearrangements (Table 4) [41,42,43,44].

Although most cases of pigmented purpuric dermatoses follow a benign course, patients may rarely subsequently develop lesions diagnostic of mycosis fungoides, and therefore, clinical follow-up is recommended, particularly in cases with widespread or persistent disease [45].

## 6. Psoriasis

Psoriasis is a chronic immune-mediated dermatosis that in atypical, long-standing, or treatment-resistant presentations may clinically and histopathologically mimic CTCL, particularly MF, and conversely, may be mimicked by MF, resulting in frequent diagnostic confusion [14,15,16].

Recognition of neutrophilic parakeratosis, absence of disproportionate epidermotropism, lack of lymphocyte atypia, and appropriate therapeutic response is essential for distinguishing psoriasis from MF (Table 5) [14,15,16].

## 7. Morphea

Early morphea, particularly in its inflammatory stage, represents an important diagnostic pitfall, as it may closely mimic CTCL, especially MF, both clinically and histopathologically [46]. Several reports have demonstrated that morphea can exhibit band-like lymphocytic infiltrates, epidermotropism, and even interstitial patterns resembling CTCL, including patch-stage and interstitial variants [12]. In some cases, the histopathologic overlap may be striking, with features such as dense dermal lymphoid infiltrates and interface changes raising concern for lymphoma [47]. This overlap has been described in both localized and generalized forms of morphea, further complicating the diagnostic process [12,47]. These findings underscore the importance of careful clinicopathologic correlation and awareness of this entity to avoid misdiagnosis and inappropriate management [46,47].

## 8. Lichen Planus/Lichenoid Dermatitis

**Figure 5:**
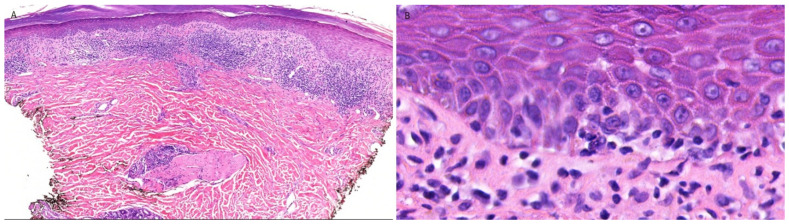
(**A**,**B**) Pseudolymphomatous lichen striatus exhibiting lichenoid interface dermatitis with peri-eccrine involvement. This figure is from the corresponding author’s laboratory and illustrates the pathological concept of typical pseudolymphomatous lichen striatus.

**Figure 6:**
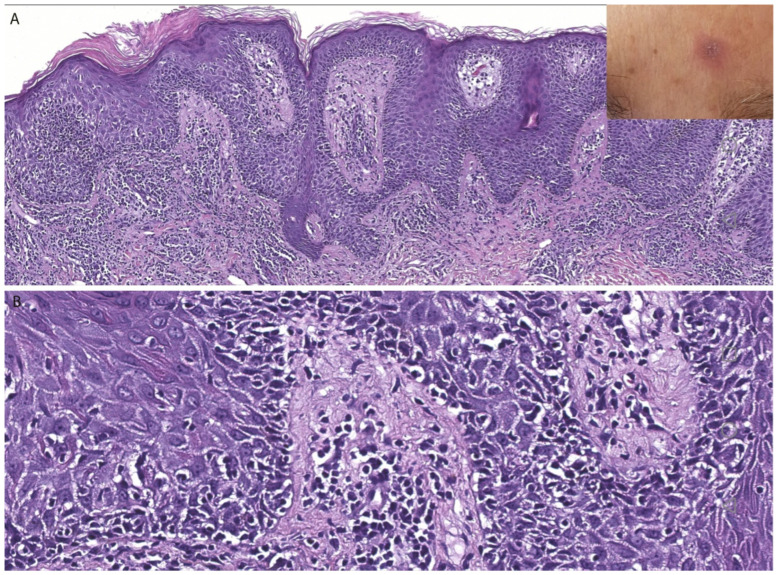
(**A**,**B**) Lichen planus-like keratosis. Inset showing the clinical presentation of a solitary erythematous papule on trunk. This figure is from the corresponding author’s laboratory and illustrates the pathological concept of typical pseudolymphomatous lichen planus-like keratosis.

Lichen planus (LP) is a chronic T-cell-mediated inflammatory dermatosis that, particularly in its hypertrophic, adnexotropic, or atypical variants, may demonstrate pseudolymphomatous histopathologic features mimicking CTCL, most notably MF, creating a recognized diagnostic pitfall. Overlap with entities such as lichen striatus (LS) (Figure 5) and lichen planus-like keratosis (Figure 6) further broadens the morphologic spectrum, and several reports document LP or LS initially misinterpreted as lymphoma due to dense infiltrates, adnexotropism, or unusual cytotoxic phenotypes [11,13,48].

Recognition of classic interface dermatitis, epidermal hypergranulosis, lack of significant lymphocyte atypia, and absence of reproducible immunophenotypic or molecular aberrations, together with appropriate clinical distribution, is essential for distinguishing pseudolymphomatous LP from CTCL (Table 6) [11,13,48].

## 9. Pityriasis Lichenoides

Pityriasis lichenoides (PL) is an inflammatory dermatosis encompassing a clinical spectrum from pityriasis lichenoides et varioliformis acuta to pityriasis lichenoides chronica, which may exhibit pseudolymphomatous histopathologic features overlapping with CTCL, particularly MF, creating a recognized diagnostic challenge [49,50,51,52,53].

Distinction of PL from MF requires the integration of clinical evolution with crops of lesions, wedge-shaped infiltrates with epidermal necrosis, cytotoxic CD8-positive immunophenotypes, and non-progressive clinical behavior, as molecular clonality alone does not reliably distinguish these entities (Table 7) [49,50,51,52,53].

## 10. Annular Lichenoid Dermatitis of Youth

**Figure 7:**
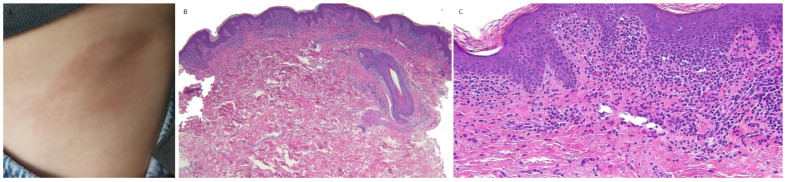
(**A**–**C**) Annular lichenoid dermatitis of youth. (**A**) Clinical presentation, (**B**,**C**) histology exhibiting dense focally lichenoid infiltrate centered at rete ridge tips. This figure is from the corresponding author’s laboratory and illustrates the pathological concept of typical pseudolymphomatous annular lichenoid dermatitis of youth.

Annular lichenoid dermatitis of youth is an uncommon inflammatory dermatosis primarily affecting children and adolescents that may mimic CTCL, particularly hypopigmented or annular variants of MF, constituting a recognized diagnostic pitfall (Figure 7) [54,55,56,57].

Recognition of keratinocyte apoptosis confined to rete ridge tips, absence of cytologic atypia or papillary dermal fibrosis, polyclonal T-cell receptor gene rearrangement, and benign clinical course is essential for distinguishing the annular lichenoid dermatitis of youth from early MF and avoiding the overdiagnosis of CTCL (Table 8) [54,55,56,57].

## 11. Contact Dermatitis

**Figure 8:**
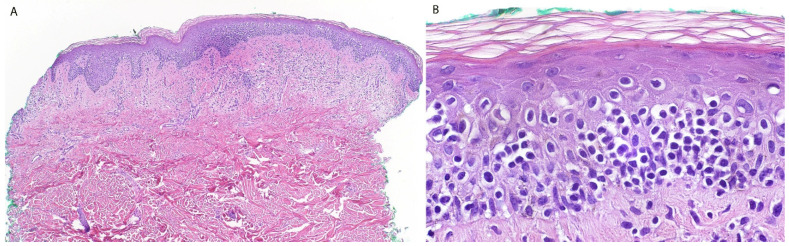
(**A**,**B**) Pseudolymphomatous contact dermatitis. This figure is from the corresponding author’s laboratory and illustrates the pathological concept of typical pseudolymphomatous contact dermatitis.

Contact dermatitis, particularly lymphomatoid contact dermatitis, is a delayed-type hypersensitivity reaction that may develop pseudolymphomatous histopathologic features closely mimicking CTCL/MF, resulting in significant diagnostic difficulty (Figure 8) [17,18,19,20,58].

Common causes of contact dermatitis include rubber compounds, nickel, methylchoroisothiazolinone/methylisothiazolinone, topical medications, and paraphenylenediamine correlation [59]. Correlation of exposure history, presence of spongiosis and eosinophils, preserved T-cell immunophenotype, and rapid clinical improvement following allergen avoidance is essential for distinguishing lymphomatoid contact dermatitis from MF/CTCL (Table 9) [17,18,19,20,58,59,60,61].

Hyperkeratotic palmoplantar eczema represents an additional important clinical and histopathologic mimicker of MF, particularly in acral sites. Histologically, features such as clustered intraepidermal lymphocytes and Langerhans cell microgranulomas favor an eczematous process over MF [60].

## 12. Actinic Reticuloid

Actinic reticuloid, also called chronic actinic dermatitis, is a severe photosensitive inflammatory dermatosis that may demonstrate pseudolymphomatous histopathologic features closely mimicking CTCL/MF [3,62,63,64].

Integration of marked photosensitivity, eczematous epidermal changes, CD8-predominant immunophenotype, and polyclonal molecular findings, together with clinical phototesting when available, is essential for distinguishing actinic reticuloid from MF/CTCL (Table 10) [3,62,63,64,65].

## 13. Arthropod Bite Reactions

Arthropod bite reactions may occasionally elicit a pseudolymphomatous cutaneous lymphoid infiltrate that mimics CTCL and represent a well-recognized cause of diagnostic confusion within the spectrum of cutaneous pseudolymphomas [66,67,68,69,70]. In such cases, including tick bites, deeper histologic sections may reveal the inciting associated changes, highlighting the importance of thorough tissue evaluation and clinicopathologic correlation [70]. They may more frequently mimic other cutaneous lymphoproliferative disorders such as lymphomatoid papulosis or primary cutaneous CD4-positive small/medium T-cell lymphoproliferative disorder. Nonetheless, overlap with MF may arise in cases with dense lymphoid infiltrates and limited epidermotropism, warranting careful clinicopathologic correlation [71].

Recognition of a polymorphous inflammatory infiltrate rich in eosinophils, together with appropriate clinical distribution, preserved immunophenotype, and benign clinical evolution, is essential for distinguishing arthropod bite reactions from CTCL (Table 11) [66,67,68,69,70].

## 14. Drug Reactions

Pseudolymphomatous drug reactions represent benign, medication-induced lymphoid proliferations that clinically and histopathologically resemble cutaneous lymphomas, particularly MF. These reactions may arise in classic drug-induced pseudolymphoma syndromes or within variants such as fixed drug eruptions (FDEs), where dense epidermotropic lymphoid infiltrates can simulate MF. Clinically, patients may present with persistent or recurrent patches or plaques that may be localized or generalized, showing a chronicity, distribution pattern, or morphology that overlaps with early CTCL [22,72,73]. The latency between drug initiation and eruption is variable, typically ranging from days to several weeks, although longer intervals have been reported, while resolution is generally observed within weeks after discontinuation of the offending agent [58].

Histopathologically, drug eruptions exhibit a lichenoid or interface dermatitis with basal vacuolar degeneration, necrotic keratinocytes, and a superficial perivascular lymphocytic infiltrate, often with eosinophils or neutrophils. FDEs, in particular, demonstrate recurrent lesions at identical sites upon re-exposure and may show intraepidermal vesiculation, pigment incontinence, and a CD8-predominant epidermotropic infiltrate. The localization and persistence of intraepidermal CD8+ memory T cells explain both the fixed nature of these eruptions and their histologic resemblance to CD8+ lymphoproliferative disorders, including MF mimickers. Such overlap has been highlighted in reported cases of generalized FDE, including amlodipine-triggered disease, where plaques were clinically indistinguishable from MF prior to drug withdrawal [22]. In addition to FDE, commonly implicated agents in pseudolymphomatous reactions include anticonvulsants (e.g., phenytoin, carbamazepine), antibiotics, antihypertensives, and biologic agents [58].

The pseudolymphomatous variant is identified when the inflammatory infiltrate becomes sufficiently dense, band-like, epidermotropic, or cytologically atypical to simulate MF. Drug-induced pseudolymphoma may demonstrate Pautrier-like collections, cerebriform lymphocytes, and either CD4- or CD8-predominant phenotypes that closely parallel CTCL. Importantly, T-cell receptor gene rearrangement studies may show clonality, which does not exclude a drug etiology. Clonality has been repeatedly documented in reactive drug eruptions, especially in FDE, and must therefore be interpreted with clinical correlation rather than used alone to establish malignancy [72]. Cases of multifocal or generalized FDE induced by drugs such as NSAIDs or amlodipine have closely mimicked MF before the drug trigger was identified [22,72,73].

Drug reaction with eosinophilia and systemic symptoms (DRESS), also referred to as drug-induced hypersensitivity syndrome, may also present with dense lymphoid infiltrates and atypical lymphocytes, occasionally mimicking CTCL both clinically and histopathologically [74].

Additionally, rare MF-like lymphomatoid reactions have been reported following vaccination, including COVID-19 vaccines [75].

Features supporting a drug-related process include a clear temporal relationship between medication exposure and lesion appearance, recurrence of identical lesions with re-challenge, presence of eosinophils or neutrophils within the infiltrate, and complete or near-complete resolution after discontinuation of the suspected agent [72,73].

## 15. Tattoo-Related Pseudolymphoma

Tattoo-related pseudolymphoma refers to a benign lymphoid proliferation that develops within a tattoo, typically months to years after placement. Clinically, these lesions often arise as pruritic or asymptomatic erythematous plaques or nodules restricted to specific pigment colors, most notably red, which commonly contains mercury sulfide. Although most cases are localized, they may cause significant diagnostic concern due to their resemblance to cutaneous lymphomas and their sometimes indolent but persistent clinical course [76].

Typical histopathology shows a dense lymphoid infiltrate involving the upper and mid-dermis, often with a top-heavy pattern and a preserved or partial grenz zone. Epidermal changes may include acanthosis, parakeratosis, spongiosis, or focal interface dermatitis. Pigment granules (commonly red or black) are frequently identified. The infiltrate may display a mixture of small and medium lymphocytes with scattered histiocytes and plasma cells, and granulomatous aggregates can be present. Immunophenotyping often reveals a predominance of T lymphocytes (CD2+, CD3+, and CD4+), though B-cell-rich patterns also occur. Importantly, T-cell-predominant lesions remain polyclonal, supporting a reactive process [23,24,76]. In some cases, atypical lymphocytes may express CD30, raising a differential diagnosis of lymphomatoid papulosis [2].

The pseudolymphomatous variant becomes diagnostically challenging, because its dense, sometimes atypical lymphoid infiltrate and epidermotropism may simulate CTCL. Loss of pan-T markers such as CD7, irregular nuclear contours, and infiltration between collagen bundles can further mimic lymphoma histologically [23]. However, several key features favor pseudolymphoma, like the presence of abundant tattoo pigment within the infiltrate, confinement of inflammation to the pigmented areas, especially red pigment, and a polyclonal lymphoid population on molecular studies [23,24]. The latency between tattooing and lesion onset can range from months to decades, with occasional reports of flares triggered by sunlight or sweating, underscoring the role of delayed hypersensitivity reactions to metallic pigments [23,24,76].

## 16. Pseudolymphomatous Folliculitis

**Figure 9:**
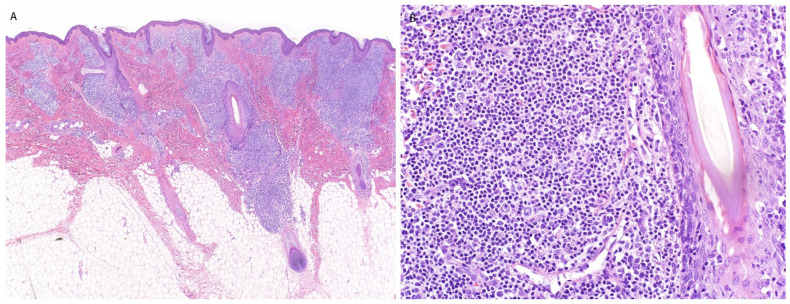
(**A**,**B**) Pseudolymphomatous folliculitis exhibiting dense folliculocentric lymphoid infiltrate. This figure is from the corresponding author’s laboratory and illustrates the pathological concept of typical pseudolymphomatous folliculitis.

Folliculitis is an inflammatory disorder of the hair follicle that clinically presents as follicular papules or nodules and histologically as perifollicular and intrafollicular inflammatory infiltrates without cytologic atypia [77]. Pseudolymphomatous folliculitis (PLF) is a rare benign cutaneous pseudolymphoma characterized by a dense lymphoid infiltrate centered on and invading hair follicles, often presenting as a solitary papule or nodule on the face, scalp, or nose. An initial case series established PLF as a distinct entity defined by a deep, folliculocentric, polymorphous lymphoid infiltrate that closely mimics cutaneous lymphoma but lacks cytologic features (Figure 9) [78]. A rare variant of follicular mycosis fungoides can elicit comedones, clinically and histologically, whereas this feature is absent in pseudolymphomatous folliculitis [79]. Subsequent reports expanded the morphologic spectrum of PLF, demonstrating that lesions may show marked folliculotropism and even focal epidermotropism [80,81,82].

Distinction from CTCL, particularly folliculotropic MF, relies on architectural, cytologic, immunophenotypic, and molecular features. PLF shows a polymorphous infiltrate with the preservation of follicular structures, retention of pan–T-cell antigens, and polyclonal T-cell receptor gene rearrangements [78,80,81]. In contrast, CTCL demonstrates monomorphic atypical lymphocytes with cerebriform nuclei, true follicular epidermotropism, aberrant antigen loss (e.g., CD7), and clonal T-cell receptor rearrangements [83].

## 17. Syphilis

Syphilis is a well-known clinical and histologic imitator of CTCL, and both primary and secondary stages may closely resemble cutaneous or mucosal lymphoproliferative disorders. Typical lesions show dense lymphoplasmacytic infiltrates in lichenoid or perivascular patterns, and this inflammatory richness, especially when plasma cells are abundant, can create concern for lymphoma [84]. Unusual presentations such as oral ulcerations or nodular secondary syphilis intensify this difficulty, as these lesions may be persistent, indurated, or architecturally complex. Several reports have highlighted that secondary and malignant syphilis, particularly in the setting of human immunodeficiency virus (HIV) coinfection, may closely mimic cutaneous T-cell lymphoma both clinically and histologically, with features including epidermotropism, CD8-predominant infiltrates, cytologic atypia, and even monoclonal T-cell receptor rearrangements, which emphasizes that clonality and atypia do not exclude an infectious etiology and must be interpreted alongside serologic and clinical data [85,86,87,88].

Histologically, syphilis may display dense lymphoid aggregates, germinal-center-like structures, or prominent epidermotropism, all of which may mimic MF or B-cell lymphomas. Nodular secondary syphilis has been documented with cerebriform lymphocytes, folliculotropism, and band-like infiltrates that strongly resemble CTCL. Yet the polyclonal nature of the infiltrate, mixed B- and T-cell populations, and especially the presence of numerous plasma cells help distinguish syphilis from true lymphoma [3,25].

Distinguishing syphilis-induced pseudolymphoma from true lymphoma requires careful clinicopathologic correlation. Polyclonality of plasma cells, equal κ and λ light-chain expression, and the presence of both CD20+ B cells and CD3+ T cells contrast with the monotypic or aberrant profiles seen in neoplastic lymphoproliferations. The identification of *Treponema pallidum* by immunohistochemistry or serologic confirmation is critical, especially when histology is misleading, as serologic tests may be initially nonreactive in primary disease or affected by the prozone phenomenon [25,84]. Importantly, prompt resolution of lesions following penicillin therapy strongly supports a syphilitic pseudolymphomatous process [3,25,84].

## 18. Borrelia

Borrelial lymphocytoma is a cutaneous pseudolymphoma triggered by *Borrelia burgdorferi* and presents as a solitary red violaceous nodule or plaque, most often on the nipple, earlobe, or genital area in endemic regions [27]. It usually appears weeks to months after a tick bite and typically resolves completely after appropriate antibiotic therapy. Although lesions may arise at the site of the initial tick bite, they are not strictly confined to it and can occur at distant cutaneous locations, reflecting the disseminated nature of Borrelia infection [89].

Histopathology shows dense dermal lymphoid infiltrates with prominent germinal centers, which may appear blastic, confluent, or lack mantle zones, which are architectural patterns that can resemble B-cell lymphoma (). Plasma cells and eosinophils are commonly present, and germinal centers characteristically express CD10 and BCL6 while lacking BCL2, a profile favoring a reactive rather than neoplastic process [27]. In addition to the classic B-cell-rich infiltrates, cutaneous borreliosis may also present with T-cell-predominant infiltrates that show epidermotropism, cytologic atypia, and band-like patterns, raising concern for MF [30]. However, the presence of mixed inflammatory cells, polytypic plasma cells, tingible body macrophages, and appropriate anatomic distribution strongly support a reactive Borrelia-associated process [27,30].

Detection of *Borrelia* may aid in confirming the diagnosis. Serologic testing and tissue-based methods such as polymerase chain reaction (PCR) can be helpful; however, the organism is not always demonstrable, and a negative result does not exclude the diagnosis in the appropriate clinicopathologic context [30].

## 19. Molluscum Contagiosum, Milker’s Nodules, and Orf

Molluscum contagiosum (MC) may rarely trigger a pseudolymphomatous reaction. Typical MC shows a lobulated endophytic proliferation with molluscum bodies in keratinocytes, but in atypical presentations, especially in older adults, unusual locations, or inflamed lesions, the dermal response may be unusually dense [90]. Recognition of molluscum bodies is the key diagnostic clue. The pseudolymphomatous variant of MC typically shows a predominance of CD3+ T cells, often CD4-biased, with scattered CD20+ cells and occasional CD30 expression [26,90]. Despite alarming atypia, clonal TCR rearrangements are consistently absent, and lesions resolve after excision or spontaneously, supporting their reactive nature.

Milker’s nodules, caused by *Parapoxvirus* (pseudocowpox), are zoonotic infections producing solitary or multiple tender nodules on the hands after exposure to infected cattle. Histopathology shows epidermal necrosis, ballooning degeneration, and a dermal mixed infiltrate composed of lymphocytes, histiocytes, and eosinophils. Rare reports document exuberant lymphoid hyperplasia with atypical lymphocytes simulating lymphoma, but absence of clonality and the presence of viral cytopathic changes favor a reactive interpretation [91]. Lesions resolve spontaneously within 4–6 weeks, supporting their benign nature.

Orf (ecthyma contagiosum), caused by the related *Parapoxvirus* of sheep and goats, can trigger pseudolymphoma. Orf-related pseudolymphoma presents as persistent nodules with a dense dermal lymphocytic infiltrate, often with germinal center formation and features resembling cutaneous B-cell pseudolymphoma. Histopathology may show epidermal acanthosis, ballooning degeneration, and viral inclusions, but deeper sections reveal lymphoid follicles with polyclonal B cells and mixed T cells [72]. The key distinguishing features are the presence of viral changes in the epidermis, a polymorphous and polyclonal infiltrate, and complete resolution over weeks.

## 20. Herpetic Infections

Herpes simplex virus (HSV-1/2) and varicella zoster virus (VZV) can occasionally produce atypical lesions that lack vesicles and instead present as plaques or nodules that are clinically suspicious for lymphoma. In these situations, biopsies may reveal dense lymphoid infiltrates with cytologic atypia that closely mimic CTCL or CD30+ lymphoproliferative disorders [28,29,92,93].

The typical histopathology of herpes infections includes ballooning degeneration, necrotic keratinocytes, and multinucleated keratinocytes with margination, but in atypical cases, these epidermal changes may be subtle or absent. The pseudolymphomatous variant is characterized by dense, often superficial, and deep perivascular and periadnexal lymphoid infiltrates with atypical lymphocytes, angiotropism or angiodestruction, and at times, extension into the subcutis [28,92]. Immunohistochemically, these infiltrates are typically T-cell-predominant (CD3+) and often CD4-skewed, with variable numbers of CD30+ and CD56+ cells; in some cases, CD30+ cells form clusters that are virtually indistinguishable from CD30+ lymphoproliferative disorders [28,92]. Rarely, the PCR for T-cell receptor genes reveals a monoclonal band [28]. Cases of herpes folliculitis with dense atypical infiltrates and CD56+ cells have been initially interpreted as angiocentric lymphoma before deeper or additional sections revealed herpetic cytopathic changes [29,92].

## 21. Scabies

Scabies, especially its nodular form, can mimic cutaneous lymphoma [3]. On routine histology, typical scabies show epidermal spongiosis, parakeratosis, eosinophils, and sometimes mites in the stratum corneum. However, nodular lesions instead reveal a deep, dense, T-cell-rich infiltrate with eosinophils and plasma cells, occasionally accompanied by epidermotropism or Pautrier-like collections [3,94]. In some cases, activated large atypical lymphocytes may express CD30, raising concern for lymphomatoid papulosis (LyP) or other CD30+ lymphoproliferative disorders [95].

Molecular studies are generally polyclonal, though occasional oligoclonal or faint clonal T-cell receptor rearrangements have been reported [3,69,95]. While the identification of mites or their products can be helpful, particularly with deeper sections, nodular scabies lesions often represent a hypersensitivity reaction, and the organism may not be demonstrable [95].

Distinguishing scabies-associated pseudolymphoma requires recognizing a mixed infiltrate with eosinophils and plasma cells and integrating clinical clues such as intense pruritus, affected household members, or lesions in genital or axillary regions, and/or improvement after scabicidal therapy [3,69,95].

## 22. Nevoid Hyperkeratosis of the Nipple/Areola

Nevoid hyperkeratosis of the nipple and areola (NHNA) is a rare benign disorder presenting as verrucous or velvety plaques on the nipple–areolar complex, most commonly in women, and often associated with hormonal changes such as puberty, pregnancy, or estrogen exposure [96,97]. Histopathology typically reveals marked hyperkeratosis, papillomatosis, acanthosis, and keratin plugging with only a mild superficial dermal infiltrate [96,97].

Rarely, NHNA exhibits epidermotropic lymphocytes with a CD3+- and CD4-predominant immunophenotype and partial CD7 loss [96,97,98]. The superficial dermal sclerosis, thickened collagen, and stellate fibroblasts sometimes observed in NHNA [96,97] can further complicate the picture by resembling fibrosing variants of CTCL [96,99]. However, key distinguishing features include the preservation of epidermal architecture [96,98], lymphocytes lacking significant cytologic atypia [96,99], polyclonal or indeterminate TCR gene rearrangement patterns [96,99], and the strict anatomic confinement of lesions to the nipple–areolar complex.

## 23. Conclusions

CTCLs, especially MF, represent one of the most challenging diagnoses in dermatology, because their presentations substantially histologically overlap with a wide spectrum of benign inflammatory and infectious dermatoses. This review underlines the crucial importance of clinicopathologic correlation and the judicious use of immunohistochemistry and molecular studies to distinguish CTCL from the other entities that mimic it, including multiple common autoimmune and infectious diseases.

## Figures and Tables

**Table 1:** Pseudolymphomatous cutaneous lupus erythematosus: MF-mimicking features and key distinguishing clues.

Category	Findings in Pseudolymphomatous DLE	Findings in Cutaneous Lymphoma (MF)
Clinical context	Well-demarcated erythematous plaques evolving to atrophic scarred plaques with dyspigmentation	Patches/plaques on trunk and sun-protected areas; chronic, progressive course
Architectural pattern	Nodular/diffuse or band-like infiltrates; angiocentric/perivascular patterns	Band-like superficial dermal infiltrate with epidermotropism; may show adnexotropism
Epidermotropism	Variable, usually mild and focal	Disproportionate epidermotropism, often without significant spongiosis
Cytologic atypia	Minimal to absent	Atypical lymphocytes with cerebriform nuclei
Interface change	Vacuolar alteration (may be focal)	Typically absent or minimal interface change
Dermal mucin	Increased	Typically absent
Basement membrane	Thickened	Typically not thickened
Innate immune cells	Prominent clustered plasmacytoid dendritic cells (pDCs), often in aggregates (commonly ≥ 10 cells)	pDCs may be present but typically not in dense clusters
Immunophenotype	Almost preserved pan–T-cell antigens	Aberrant phenotype with loss of pan–T-cell markers (e.g., CD7 loss; >50% loss of CD2/CD5)
Molecular studies	Polyclonal T-cell receptor (TCR) gene rearrangement	Clonal T-cell receptor (TCR) gene rearrangement

**Table 2:** Pseudolymphomatous lichen sclerosus: MF-mimicking features and key distinguishing clues.

Category	Findings in Pseudolymphomatous LS	Findings in Cutaneous Lymphoma (MF)
Clinical distribution	Predominantly anogenital; less often extragenital	Typically trunk and proximal extremities; genital involvement uncommon
Architectural pattern	Dense band-like or lichenoid infiltrate	Band-like superficial dermal infiltrate with epidermotropism; may show patchy or diffuse involvement
Epidermotropism	Present, usually focal and confined to lower epidermis	Disproportionate epidermotropism, often involving full epidermal thickness
Cytologic atypia	Mild or absent	Atypical lymphocytes with cerebriform nuclei
Interface change	Vacuolar basal alteration	Typically absent or minimal
Papillary dermis	Hyalinization and sclerosis	Papillary dermal fibrosis with wiry (“fettucine-like”) collagen bundles
Basement membrane	Thickened	Typically not thickened
Immunophenotype	Mixed T-cell population with preserved pan–T-cell markers	Aberrant phenotype with loss of pan–T-cell markers (commonly CD7; may involve CD2/CD5)
Antigen expression	Partial CD5 or CD7 reduction may occur	More extensive and reproducible antigen loss, especially in epidermotropic lymphocytes
Molecular studies	Polyclonal or occasional monoclonal TCR-γ rearrangement	Clonal T-cell receptor (TCR) gene rearrangement
Clinical behavior	Stable or slowly progressive lesions; atrophy more common	Chronic, progressive evolution with potential stage progression

**Table 3:** Pseudolymphomatous vitiligo: MF-mimicking features and key distinguishing clues.

Category	Findings in Pseudolymphomatous Vitiligo	Findings in Cutaneous Lymphoma (MF)
Clinical presentation	Sharply demarcated depigmented patches, often with erythematous inflammatory rim	Hypopigmented or erythematous patches, often ill-defined with gradual progression
Lesion stage	Early or inflammatory lesions	Persistent, slowly progressive lesions
Architectural pattern	Superficial band-like or peri-junctional lymphoid infiltrate	Band-like infiltrate with epidermotropism; may show patchy or diffuse involvement
Epidermotropism	Prominent lymphocytic exocytosis, occasional Pautrier-like clusters	Disproportionate epidermotropism with formation of true Pautrier microabscesses
Cytologic atypia	Minimal to absent	Atypical lymphocytes with cerebriform nuclei
Melanocytes	Reduced or absent melanocytes on Melan-A/HMB-45 staining	Typically preserved melanocytes (may be decreased in hypopigmented MF but not completely absent)
Interface change	Mild vacuolar alteration	Typically absent or minimal
Immunophenotype	Mixed CD4/CD8 T-cell infiltrate, often CD8predominant	Classically CD4predominant; CD8 predominance may be seen in hypopigmented MF
Pan–T-cell markers	Preserved	Aberrant phenotype with loss of pan–T-cell markers (commonly CD7)
Innate immune features	Increased plasmacytoid dendritic cells (pDCs) with type I interferon-associated activity, including expression of interferon-inducible markers (e.g., MxA) and clustering at the dermoepidermal junction, particularly at lesion margins	pDCs may be present but typically lack prominent clustering at the dermoepidermal junction and do not show a strong type I interferon-associated signature
Molecular studies	Polyclonal T-cell receptor (TCR) gene rearrangement	Clonal T-cell receptor (TCR) gene rearrangement
Clinical course	Repigmentation or lesion stabilization	Persistent and progressive disease course

**Table 4:** Pseudolymphomatous pigmented purpuric dermatoses/lichen aureus: MF-mimicking features and key distinguishing clues.

Category	Findings in Pseudolymphomatous PPD/Lichen Aureus	Findings in Cutaneous Lymphoma (MF)
Clinical presentation	Solitary or localized golden brown to violaceous patches or plaques	Erythematous patches or plaques, often multiple and progressive
Anatomic distribution	Predominantly lower extremities	Often trunk and proximal extremities; may become widespread
Architectural pattern	Dense band-like or deep dermal lymphoid infiltrate	Band-like superficial dermal infiltrate with epidermotropism
Epidermotropism	May be present, usually focal	Disproportionate epidermotropism, often more extensive
Cytologic atypia	Mild or absent	Atypical lymphocytes with cerebriform nuclei
Dermal features	Erythrocyte extravasation and hemosiderin deposition	Typically absent
Epidermal changes	Lichenoid/interface changes may be present in lichen aureus variant and the lichenoid variant	Epidermotropism with atypical lymphocytes; minimal spongiosis
Immunophenotype	Predominantly T-cell infiltrate, often CD4-skewed	Typically CD4-predominant with aberrant phenotype
Pan–T-cell markers	Preserved; partial CD7 reduction may occur	Aberrant phenotype with reproducible loss of pan–T-cell markers (e.g., CD7)
Molecular studies	Occasional monoclonal TCR-γ rearrangement; identical (matching) clones across lesions or may be observed over time but can occur in reactive processes and are not diagnostic of MF	Clonal T-cell receptor (TCR) gene rearrangement
Clinical course	Usually stable, non-progressive; rare progression to MF reported, particularly in widespread or atypical cases	Chronic, progressive disease course

**Table 5:** Psoriasis as a mimicker of mycosis fungoides: overlapping and distinguishing features.

Category	Findings in Psoriasis Mimicking CTCL	Findings in Cutaneous Lymphoma (MF)
Clinical presentation	Persistent erythematous or hyperkeratotic plaques	Erythematous patches or plaques with gradual progression
Anatomic sites	Palmoplantar or solitary plaques common	Typically trunk and proximal extremities; may become widespread
Architectural pattern	Psoriasiform epidermal hyperplasia	Band-like superficial dermal infiltrate with epidermotropism; no true psoriasiform hyperplasia
Epidermal scale	Confluent parakeratosis	Minimal or absent scale
Granular layer	Reduced or absent	Usually preserved
Neutrophils	Munro microabscesses and spongiform pustules of Kogoj	Typically absent
Dermal infiltrate	Mild superficial perivascular lymphocytes	Dense band-like lymphoid infiltrate in superficial dermis
Epidermotropism	Absent or minimal	Disproportionate epidermotropism with atypical lymphocytes
Cytologic atypia	Absent	Atypical lymphocytes with cerebriform nuclei
Immunophenotype	Preserved pan–T-cell antigen expression	Aberrant phenotype with loss of pan–T-cell markers (e.g., CD7)
Molecular studies	No consistent T-cell receptor (TCR) clonality	Clonal T-cell receptor (TCR) gene rearrangement
Treatment response	Improvement with appropriate antipsoriatic therapy	Persistent disease despite standard anti-inflammatory therapy

**Table 6:** Pseudolymphomatous lichen planus: MF-mimicking features and key distinguishing clues.

Category	Findings in Pseudolymphomatous Lichen Planus	Findings in Cutaneous Lymphoma (MF)
Clinical presentation	Violaceous, polygonal papules or plaques; hypertrophic or linear variants	Erythematous patches, plaques, or tumors with gradual progression
Distribution pattern	Symmetric, flexural, or Blaschkoid involvement	Typically trunk and proximal extremities; often asymmetric or patchy
Architectural pattern	Dense band-like lichenoid infiltrate	Band-like superficial dermal infiltrate with epidermotropism
Epidermotropism	Present but limited and reactive	Disproportionate epidermotropism with atypical lymphocytes
Cytologic atypia	Absent or minimal	Atypical lymphocytes with cerebriform nuclei
Interface change	Prominent vacuolar basal alteration with Civatte bodies	Typically absent or minimal interface change
Epidermal features	Hypergranulosis and compact orthokeratosis	Usually lacks hypergranulosis; may show epidermal atrophy or mild hyperplasia
Adnexal involvement	Perifollicular or peri-eccrine inflammation without infiltration of adnexal epithelium	True syringotropism or folliculotropism with infiltration of adnexal epithelium by atypical lymphocytes
Immunophenotype	Predominantly T-cell infiltrate with preserved pan–T-cell markers	Aberrant phenotype with loss of pan–T-cell markers (e.g., CD7)
Antigen expression	No consistent loss of CD5 or CD7	Reproducible loss of pan–T-cell markers, especially CD7
Molecular studies	Polyclonal T-cell receptor (TCR) gene rearrangement (often CD8-predominant)	Clonal T-cell receptor (TCR) gene rearrangement
Clinical course	Chronic but non-progressive; treatment responsive	Chronic, progressive disease course

**Table 7:** Pityriasis lichenoides as a mimicker of mycosis fungoides: overlapping and distinguishing features.

Category	Findings in Pityriasis Lichenoides	Findings in Cutaneous Lymphoma (MF)
Clinical presentation	Recurrent crops of papules at different stages of evolution	Persistent patches or plaques with gradual progression
Disease variants	Acute (pityriasis lichenoides et varioliformis acuta) and chronic forms	No equivalent acute/chronic inflammatory spectrum
Architectural pattern	Superficial and deep wedge-shaped lymphohistiocytic infiltrate	Band-like superficial dermal infiltrate with epidermotropism
Epidermal changes	Parakeratosis, necrotic keratinocytes, focal ulceration	Typically lacks keratinocyte necrosis; may show epidermotropism with atypical lymphocytes
Epidermotropism	May be present and prominent	Disproportionate epidermotropism with atypical lymphocytes
Cytologic atypia	Mild to moderate, reactively appearing	Atypical lymphocytes with cerebriform nuclei
Dermal features	Erythrocyte extravasation and interface damage	Typically absent or minimal interface change
Immunophenotype	Predominantly CD8-positive cytotoxic T-cell infiltrate	Classically CD4-predominant; CD8 phenotype may be seen in variants
Cytotoxic markers	Expression of TIA-1 and granzyme B	May be present in cytotoxic variants of MF
Pan–T-cell markers	Preserved	Aberrant phenotype with loss of pan–T-cell markers (e.g., CD7)
Molecular studies	Monoclonal TCR-γ rearrangement in subset; clonality may be reactive	Clonal T-cell receptor (TCR) gene rearrangement
Clinical course	Relapsing–remitting, often self-limited or phototherapy-responsive; rare progression to MF reported, warranting monitoring for persistent or atypical lesions	Chronic, progressive disease course

**Table 8:** Annular lichenoid dermatitis of youth as a mimicker of mycosis fungoides: overlapping and distinguishing features.

Category	Findings in Annular Lichenoid Dermatitis of Youth (ALDY)	Findings in Cutaneous Lymphoma (MF)
Clinical presentation	Annular or oval erythematous plaques with central hypopigmentation	Hypopigmented or erythematous patches/plaques with gradual progression
Age group	Predominantly children and adolescents	Typically in adults; rare in children
Anatomic distribution	Groin, flanks, trunk	Typically trunk and proximal extremities; may be widespread
Architectural pattern	Dense lichenoid infiltrate centered at rete ridge tips	Band-like superficial dermal infiltrate with epidermotropism
Epidermotropism	Present but reactive and limited	Disproportionate epidermotropism with atypical lymphocytes
Cytologic atypia	Mild, sometimes present	Atypical lymphocytes with cerebriform nuclei
Keratinocyte apoptosis	Prominent apoptosis confined to rete ridge tips	Typically absent or not restricted to rete ridge tips
Papillary dermis	Lacks papillary dermal fibrosis	Papillary dermal fibrosis often present in longstanding lesions
Immunophenotype	Predominantly CD8-positive epidermotropic T cells with dermal CD4-positive cells	Classically CD4-predominant; CD8 variants may occur
Langerhans cells	Increased suprabasal Langerhans cells	Typically not increased in this pattern
Molecular studies	Polyclonal T-cell receptor (TCR) gene rearrangement	Clonal T-cell receptor (TCR) gene rearrangement
Clinical course	Benign, often responsive to topical therapy or self-limited	Chronic, progressive disease course

**Table 9:** Lymphomatoid contact dermatitis as a mimicker of mycosis fungoides: overlapping and distinguishing features.

Category	Findings in Lymphomatoid Contact Dermatitis	Findings in Cutaneous Lymphoma (MF)
Clinical presentation	Chronic pruritic patches or plaques at sites of allergen exposure	Erythematous patches or plaques with gradual progression, not restricted to exposure sites
Common locations	Buttocks, thighs, groin, areas of repeated exposure	Typically trunk and proximal extremities; may become widespread
Temporal relationship	Onset or persistence linked to allergen exposure	No clear external trigger; persistent and progressive
Architectural pattern	Dense band-like or nodular dermal lymphoid infiltrate	Band-like superficial dermal infiltrate with epidermotropism
Epidermotropism	Prominent lymphocytic exocytosis, occasional Pautrier-like collections	Disproportionate epidermotropism with atypical lymphocytes and true Pautrier microabscesses
Cytologic atypia	Mild, reactive-appearing; lymphocytes are small to medium-sized with round to slightly irregular nuclei, lacking true cerebriform contours, often with a mixed population including stretched or small, round lymphocytes	Atypical lymphocytes with cerebriform nuclei
Spongiosis	Present; may be subtle in chronic lesions	Typically absent or minimal
Eosinophils	Often present within infiltrate	Typically absent in early MF but may be present in some cases, particularly in advanced disease or treatment-associated settings
Langerhans cells	Langerhans cell microgranulomas may be present	Typically absent
Immunophenotype	CD4- or CD8-predominant T-cell infiltrate with preserved pan–T-cell markers	Aberrant phenotype with loss of pan–T-cell markers (e.g., CD7)
Antigen expression	Partial CD7 loss may occur	More extensive and reproducible loss of pan–T-cell markers
Molecular studies	Polyclonal or transient TCR rearrangement; clonality may be reactive	Clonal T-cell receptor (TCR) gene rearrangement
Clinical course	Marked improvement after allergen avoidance or patch testing	Persistent disease despite removal of potential triggers

**Table 10:** Actinic reticuloid as a mimicker of mycosis fungoides: overlapping and distinguishing features.

Category	Findings in Actinic Reticuloid	Findings in Cutaneous Lymphoma (MF)
Clinical presentation	Chronic eczematous, lichenified, or infiltrated plaques	Erythematous patches, plaques, or tumors with gradual progression
Photosensitivity	Severe reaction to ultraviolet A, ultraviolet B, and visible light	Typically not associated with marked photosensitivity
Distribution	Predominantly sun-exposed areas with possible extension to covered skin	Typically trunk and proximal extremities; not limited to sun-exposed areas
Architectural pattern	Dense superficial and deep lymphoid infiltrate	Band-like superficial dermal infiltrate with epidermotropism
Epidermotropism	Variable, sometimes prominent	Disproportionate epidermotropism with atypical lymphocytes
Cytologic atypia	Mild, reactive-appearing lymphocytes	Atypical lymphocytes with cerebriform nuclei
Epidermal changes	Spongiosis, acanthosis, hypergranulosis	Typically lacks significant spongiosis; may show epidermotropism with atypical cells
Dermal features	Papillary dermal fibrosis with vertically oriented collagen bundles; prominent dermal dendrocytes and multinucleated giant cells may be present	Papillary dermal fibrosis without prominent dendrocytes or giant cells
Immunophenotype	Predominantly CD8-positive T-cell infiltrate with preserved pan–T-cell markers	Classically CD4-predominant with aberrant phenotype
Antigen expression	No consistent loss of pan–T-cell antigens	Reproducible loss of pan–T-cell markers (e.g., CD7)
Molecular studies	Polyclonal T-cell receptor (TCR) gene rearrangement	Clonal T-cell receptor (TCR) gene rearrangement
Clinical course	Chronic but non-neoplastic; without progression to lymphoma	Chronic, progressive disease course

**Table 11:** Pseudolymphomatous arthropod bite reactions: MF-mimicking features and key distinguishing clues.

Category	Arthropod Bite Reaction (Pseudolymphomatous)	Mycosis Fungoides (CTCL)
Clinical presentation	Solitary or localized papules, nodules, or plaques, often with acute onset	Persistent patches or plaques with progressive evolution
Distribution	Exposed areas (face, extremities)	Often non-sun-exposed, widespread, including trunk
Temporal course	May follow insect bite; often regresses or improves spontaneously	Chronic, progressive disease
Architectural pattern	Dense superficial and/or deep dermal infiltrate	Band-like superficial infiltrate with epidermotropism
Infiltrate composition	Polymorphous: lymphocytes, eosinophils, plasma cells, histiocytes	Monomorphic atypical lymphocytes
Immunophenotype	Predominantly T-cell infiltrate with mixed inflammatory background	Typically CD4+-predominant T-cell population
Clonality	May show T-cell clonality; not diagnostic of malignancy	Clonal TCR rearrangement supports diagnosis
Cytologic atypia	Mild or reactively appearing; non-cerebriform	Atypical cerebriform lymphocytes
Epidermotropism	Usually absent or mild	Prominent and disproportionate
Eosinophils	Frequently present	Rare or minimal
Plasma cells	May be present; occasionally monotypic	Typically absent
Key diagnostic clue	Identification of arthropod or bite reaction; polymorphous infiltrate	Persistent lesions with progressive evolution
Clinical course	Partial or complete regression with conservative management	Progressive without treatment

## Data Availability

No new data were created or analysed in this study. Figure 1 demonstrates typical pseudolymphomatous LE. Figure 2 demonstrates typical pseudolymphomatous lichen sclerosus. Figure 3 demonstrates typical pseudolymphomatous vitiligo. Figure 4 demonstrates typical pseudolymphomatous PPD. Figure 5 demonstrates typical pseudolymphomatous lichen striatus. Figure 6 demonstrates typical pseudolymphomatous lichen planus-like keratosis. Figure 7 demonstrates typical pseudolymphomatous annular lichenoid dermatitis of youth. Figure 8 demonstrates typical pseudolymphomatous contact dermatitis. Figure 9 demonstrates typical pseudolymphomatous folliculitis.
